# Emerging Roles of PTTG1/Securin in Breast Cancer

**DOI:** 10.7150/jca.121650

**Published:** 2025-10-27

**Authors:** Lingyuan Min, Shaojie Feng, Xiuxiu Liu, Yan Zhang, Mengmeng Zhao, Huan Shi, Xianqiang Liu, Tianning Wang

**Affiliations:** 1 Breast Disease Diagnosis and Treatment Center/Department of Thyroid Surgery, Central Hospital Affiliated to Shandong First Medical University, Jinan, 250013, China.; 2 Research Center of Translational Medicine, Central Hospital Affiliated to Shandong First Medical University, Jinan, 250013, China.; 3 Department of Internal Medicine-Oncology, Shandong Cancer Hospital and Institute, Shandong First Medical University and Shandong Academy of Medical Sciences, Jinan, 250117, China.

**Keywords:** PTTG1, securin, breast cancer, chromosomal instability, tumor progression

## Abstract

Securin is a key regulator of chromosome segregation during mitosis. Dysregulation of securin triggers chromosomal instability (CIN) and aneuploidy, which are hallmarks of many solid tumors, including breast cancer (BC). Recent studies have revealed securin's multifaceted roles in the progression of BC. Overexpression of securin not only enhances the malignant behaviors of BC cells but also correlates with poor clinical outcomes in patients, suggesting its potential as both a therapeutic target and prognostic biomarker. Although interest in securin is growing, comprehensive reviews on its role in BC are sparse. In this review, we summarize the biological function of securin. We then focus on the expression patterns of securin in BC and related experimental models, and their association with CIN. Subsequently, we discuss the significance of securin as a prognostic marker for BC. Lastly, we explore how securin influences the malignant behaviors of BC cells. This review emphasizes the critical connection between CIN and BC pathobiology mediated by securin and offers insights for future research into securin-related mechanisms and therapeutic strategies.

## Introduction

The pituitary tumor-transforming gene (PTTG) was first isolated from GH4 rat pituitary tumor cells 1. Subsequent structural homology analyses identified PTTG1 protein as vertebrate securin, a master mitotic regulator that ensures genomic stability by controlling sister chromatid separation through the inhibition of separase activity 2. Characterized by cell cycle-dependent expression oscillations and dynamic subcellular localization, securin is a key regulator of chromosome segregation with potential roles in cell cycle control, transcriptional regulation, and DNA damage repair 3. Since these cellular events are frequently dysregulated in cancers, much effort has been invested in examining the role of securin in human tumors. In breast cancer (BC), recent data involve securin expression and mutations in clinical outcome, making it a valuable prognostic marker and therapeutic target. Research into securin's interaction networks, post-translational modifications, and subcellular trafficking continues to delineate its complex roles in carcinogenesis.

BC in females is the second leading cause of global cancer incidence in 2022, with an estimated 2.3 million new cases, accounting for 11.6% of all cancer diagnoses 4. Among women, it is the most commonly diagnosed cancer and the leading cause of cancer deaths worldwide 4. Chromosomal instability (CIN) is a characteristic of BC and is directly related to a range of clinical presentations, including disease stage, metastasis, poor prognosis, and drug resistance 5, 6. In this review, we summarize current evidence for the biological functions of securin as a prognostic marker in BC and investigate the association between securin and CIN in BC.

## The structure and function of securin

**Figure 1:**
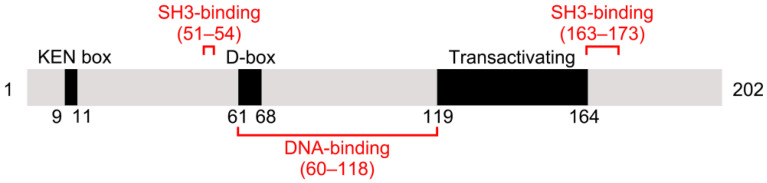
** Schematic illustration of mammalian securin protein.** Mammalian securin contains a KEN box, a D-box, and a transactivating domain. DNA-binding and SH3-binding sites are indicated in red. Positions of the elements are shown by residue numbers.

The human gene *PTTG1* is located on chromosome 5 and consists of five exons and four introns. It encodes the 202-amino acid protein securin, which is 90.6% and 89.6% identical in sequence to PTTG2 and PTTG3, respectively. Securin has an amino (N)-terminal regulatory domain and a carboxyl (C)-terminal functional domain 7. The N-terminus includes a conserved destruction box (D-box) and a KEN-box. Securin degradation is primarily regulated by the anaphase-promoting complex/cyclosome^CDC20^ (APC/C^CDC20^) through its D-box, with the KEN-box contributing less 8. Securin contains two proline-rich motifs within its C-terminus, which together with a specific domain in the N-terminus constitute a Src homology 3 (SH3)-binding site 9 (Figure 1). Securin can bind to various SH-3-proteins with this site, and that is of crucial significance to securin's biological function 10.

The most critical function of securin is to ensure faithful segregation of sister chromatids in mitosis. In human cells, replicated sister chromatids are held together by the cohesin complex from the S phase to early mitosis 11. Removal of cohesin from arms of the chromosome is mediated by Sororin and wings apart-like protein homolog in prophase 12, 13, whereas removal of cohesin from centromeres is triggered by separase 14. Securin regulates separase activity by a dual mechanism. Securin serves as a pseudosubstrate by blocking access to separase's C-terminal catalytic domain 15-17. In addition, securin promotes nuclear translocation of separase for its acquisition of proteolytic activity 18. During metaphase-anaphase transition, APC/C^CDC20^ ubiquitinates securin and releases separase to cleave centromeric cohesin 19, 20. Sister chromatids are thus accurately segregated into two daughter cells, which is essential for maintaining genomic stability. The ubiquitination level of securin is directly regulated by its own phosphorylation and acetylation status and indirectly modulated by the APC/C activity 21-26. Furthermore, RSUME extends the half-life of securin, and their co-expression leads to an increase in chromosomal abnormalities 27.

Furthermore, securin possesses intrinsic DNA-binding ability and functions as a transcription factor 9, 28. The transcription of several genes, including *c-myc*, *PKCβ-1*, *MEK1*, *MEK3*, and *HSP70*, was activated following securin overexpression 29. Furthermore, direct transcriptional control of the mitogenic and angiogenic factor *FGF-2* by securin has been documented, dependent on the C-terminal proline-rich motifs of securin 30, 31. Securin also directly regulates the expression and secretion of prolactin 32. Tong et al. analyzed the influence of securin on the transcription of 20,000 genes, discovering that the majority of genes regulated by securin were involved in cell cycle, cell metabolism, or signal transduction, thus highlighting securin's role in the regulation of various cellular processes 33. Securin is phosphorylated by MAPK, which dynamically regulates its transcriptional activation activity 34.

**Figure 2:**
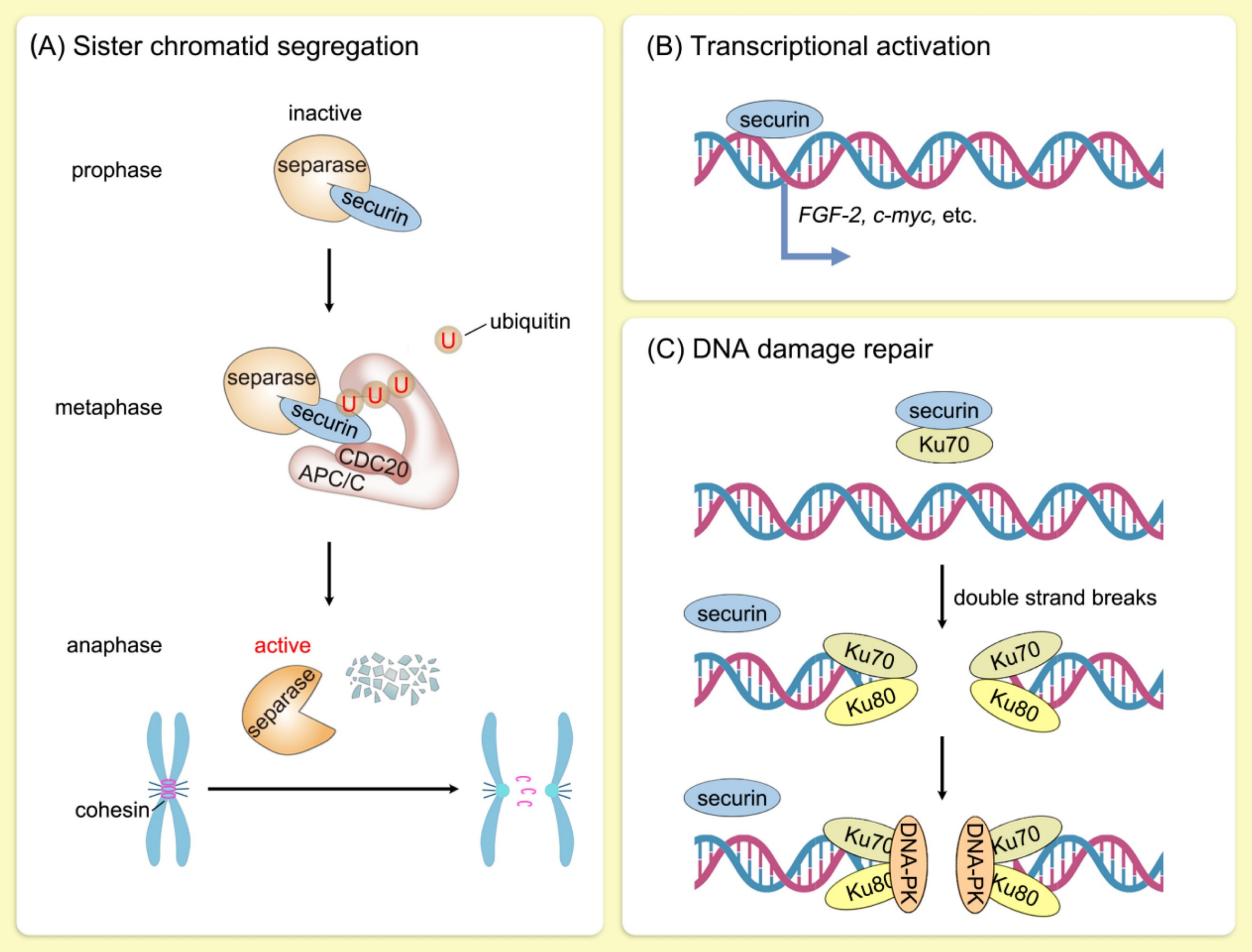
** Biological function of securin.** Schematic illustration of the biological function of securin. (A) During metaphase-anaphase transition, APC/C^CDC20^ ubiquitinates securin and releases separase to cleave centromeric cohesin. Sister chromatids are thus accurately segregated into two daughter cells, which is essential for maintaining genomic stability. U, ubiquitin. (B) Securin functions as a transcription factor. The transcription of a series of genes, including *c-myc* and *FGF-2*, was activated following securin upregulation. (C) Securin participates in DNA damage repair by interacting with Ku70, the regulatory subunit of DNA-dependent protein kinase (DNA-PK). Upon DNA-damaging events, the securin-Ku70 complex is disrupted, thereby delaying mitosis onset.

Securin was also identified as being involved in DNA damage repair. In mammalian cells, securin participates in DNA damage repair by interacting with Ku70, the regulatory subunit of DNA-dependent protein kinase (DNA-PK) 35, 36. Upon DNA-damaging events, the securin-Ku70 complex is disrupted, thereby delaying mitosis onset 35 (Figure 2). In addition, decreased securin or PBF expression led to dysregulated expression of p53 target genes involved in DNA repair and apoptosis 37. These findings further confirm the important role of securin in genomic stability.

## Securin and CIN in BC

### Expression of securin in BC and related experimental models

Numerous studies have examined the expression of securin in BC. Compared to normal tissues, securin is highly expressed in BC tissues, and is positively correlated with the tumor pathological grade 38, 39. Additionally, the level of securin expression correlates with the degree of malignancy in BC cell lines; it is more highly expressed in malignant BC cell lines compared to normal or less malignant BC cells 40, 41. Among the four subtypes of BC—HER2-positive, luminal A, luminal B, and triple-negative—securin shows higher expression in estrogen receptor (ER) negative BC tissues than in ER-positive tissues 42, with significant upregulation observed in the triple-negative breast cancer (TNBC) subgroup compared to other subgroups 43, 44. Additionally, a significant correlation between keratin 67 (Ki67) and securin was observed in invasive BC 45.

It has been reported that securin is highly expressed in mammary gland epithelial cells and is necessary for proper morphogenesis of the mammary gland in mice 46. From the early stages (4 weeks) to the later stages (13 weeks) of mammary gland development, securin is primarily expressed in the cap and body cells of the terminal end buds, as well as the single-layer luminal epithelium in the ductal region 46. Securin regulates a number of genes associated with proliferation and mammary gland branching morphogenesis. *Pttg1*-null mice displayed increased proliferation and defects in branch patterning in their mammary glands 46. Additionally, *Pttg1*-null mice exhibited testicular and splenic hypoplasia, thymic hyperplasia, and thrombocytopenia 47, indicating that securin maintains global chromosomal stability and cell cycle progression during embryonic and postnatal development. To date, no mammary gland-specific *Pttg1* knockout or transgenic mouse model has been developed, which could be valuable for further investigating the role of securin in the development and carcinogenesis of the mammary gland.

Multiple regulators of securin expression in BC have been identified. The transcription factor Oct-1 specifically bound to and transactivated the promoter of *PTTG1*, with a concurrent overexpression of Oct-1 and securin observed in BC 48. Recent studies have further demonstrated that depletion of SOS1 or KHSRP downregulated securin in BC cells 49, 50. Additionally, securin expression is modulated by certain natural hormones, such as estrogen and insulin, in MCF-7 cells 51, 52. Some clinical chemicals also regulate securin expression. For instance, statins, which are 3-hydroxyl-3-methyl glutaryl coenzyme A reductase inhibitors, have shown unpredictable benefits in reducing BC progression and mortality 53, 54. The stability of *PTTG1* mRNA was markedly decreased by several lipophilic statins in MDA-MB-231 cells, providing insights into how statins prevent BC metastasis 41. Furthermore, multiple noncoding RNAs have been predicted to modulate *PTTG1* expression, though their biological functions require further experimental verification 43, 55.

### Effects of securin on CIN in BC

CIN refers to an increased frequency of chromosomal alterations, including both numerical and structural aberrations 6. CIN enables cancer cells to rapidly increase genomic complexity through the simultaneous acquisition of small- and large-scale losses, gains, and rearrangements of DNA, and is a hallmark of cancer 56. CIN promotes tumor subclone diversification by amplifying intratumoral heterogeneity, while also enhancing phenotypic plasticity in response to selective pressures 57. Paradoxically, CIN can suppress cancer cell survival due to prolonged mitotic duration and the generation of inviable karyotypes 58, 59. Thus, understanding how cancer cell populations reach an optimal equilibrium for CIN rate is of considerable scientific interest.

Approximately 60-80% of BC deviate from a diploid karyotype, and invasive ductal carcinomas present aneuploidy significantly more often than other types of BC 60. In terms of the receptor status, ER-negative and HER2-positive were associated with a higher degree of CIN 61-63. Among luminal BC, luminal B had a higher rate of CIN than luminal A 62. The causes of CIN are diverse, including mitotic errors, replication stress, impairment of homologous recombination, and telomere dysfunction 64. CIN is associated with a wide range of clinical features of BC including disease stage, metastasis, prognosis, and therapeutic resistance 5, 6. Although high levels of CIN have generally been linked with poor clinical outcomes in BC, some studies have found that extreme CIN correlates with a good prognosis 63, 65. This paradox implies that the relationship between CIN and BC patient prognosis is complex. Therefore, identification of primary regulators controlling CIN in BC and classification of patients based on their degree of CIN is crucial for developing new therapeutic strategies and enabling personalized therapy. Accordingly, a number of therapeutics directed against CIN-related mechanisms are under development or have already been used clinically.

Considering the crucial role of securin in ensuring accurate chromosome separation, dysregulation of securin has been identified as a potential driver of CIN. However, to date, only a few studies have investigated the association between securin and CIN in BC. Ogbagabriel et al. found that securin overexpression correlated with the degree of nuclear pleomorphism, which is associated with CIN 66. Karra et al. reported that high-level securin expression predicted an increased risk of aneuploidy in BC, highlighting the link between securin expression and aneuploidy 67, 68. Talvinen et al. found that securin positivity predicted the occurrence of aneuploid DNA content 69. Securin-induced aneuploidy was prevented by p53-dependent apoptosis in MCF-7 cells, and p53 suppresses hormone-induced tumor risk and the incidence of aneuploidy by inhibiting securin expression 70, 71. Collectively, it is confirmed that securin promotes CIN through p53 in BC, but whether securin has other p53-independent mechanisms requires further investigation. Indirect evidence is provided by research into securin's transcription activation targets, such as *FGF-2*,* c-myc*, and *SP1*, all of which have been shown to associate with CIN in cancer 72-74. Future research should concentrate on clarifying the p53-independent pathways by which securin causes CIN, perhaps through validating the cellular functions of its transcriptional targets or revealing new effectors. Additionally, exploring the therapeutic value of securin and its downstream effectors to suppress CIN and aneuploidy in BC is promising.

## Securin as a prognostic marker of BC

### Expression levels

Numerous studies have confirmed the prognostic value of securin in BC. By analyzing 90 BC and 18 normal breast tissues, Ogbagabriel et al. reported that securin overexpression correlated with the mitotic index, lymph node invasion, degree of nuclear pleomorphism, and ER-α expression 66. Talvinen et al. focused on the expression of securin in invasive BC and found that securin immunopositivity was an independent prognosticator, predicting the survival of patients based on histological type, Ki-67 proliferation status, and tumor size 75, 76. Karra et al. conducted a study on 603 BC patients and found that securin was a strong independent prognostic marker for survival 67. Through comprehensive genomic analysis of Middle Eastern female patients with BC, Colak et al. identified age-specific gene signatures while systematically investigating molecular alterations associated with cancer progression in young women. Although *PTTG1* was not incorporated into the age-specific gene subset, it was one of the 16 genes involved in tumorigenesis, invasion, and progression 77. Vihervuori et al. investigated 179 TNBC patients with complete clinical data, noting that high expression of securin was significantly associated with a low fraction of tumor-infiltrating lymphocytes and CD8^+^ T cells, suggesting that the level of securin expression might be useful to evaluate the tumor inflammatory response in TNBC 78.

Meanwhile, the prognostic value of securin has been evaluated using publicly available clinical data by various researchers. They found that BC patients with higher levels of securin exhibited significantly poorer overall survival (OS), relapse-free survival (RFS), and distant metastasis-free survival (DMFS) compared to patients with relatively lower levels of securin 38-40, 43, 44, 49, 51, 79-81. Additionally, high expression of securin was prevalent in metastatic BC tissues, suggesting its potential as a biomarker for BC metastasis 39, 82.

Furthermore, several studies have confirmed the prognostic accuracy of securin in BC when combined with other genes/proteins. Chen et al. detected circulating tumor cells (CTCs) in the peripheral blood of female BC patients using a panel of four genes: *PTTG1*, *BIRC5*,* UBCH10*, and* TK1*. They found that tumor size, histological grade, lymph node metastasis, and TNM stage were significantly correlated with the positive detection rate of the multimarker panel 83. Talvinen et al. observed that combined detection of cdc27 and securin predicted cancer death in BC 69. Karra et al. investigated 445 patients with BC and found that high expression levels of Cdc20 and securin demonstrated a 6.8-fold increased mortality risk 68. Notably, this dual protein overexpression pattern was predominantly observed in TNBC patients and was particularly associated with the subgroup exhibiting extremely short survival (on average 2.4 years) 68. Repo et al. developed a prognostic model combining securin, separase, and Cdk1 for predicting tumor size, histological grade, axillary lymph node status, and the risk of mortality, based on a study comprised of 1,135 BC patients 84. Additionally, the OncoMasTR Molecular Score (OMm) is identified as a combination of master transcriptional regulators: *FOXM1*, *PTTG1*, and *ZNF367*. OMclin1 combines OMm with nodal status, tumor size, and grade, and OMclin2 is a simpler form of OMclin1 that excludes tumor grade 85. Buus et al. reported that OMm, OMclin1, and OMclin2 were highly prognostic for early and late distant recurrence in women with early-stage ER-positive BC receiving 5 years of endocrine therapy 85. Collectively, securin expression serves as a strong prognosticator of BC outcomes.

### Single nucleotide polymorphism (SNP)

SNPs are single-base differences in the DNA sequence that occur with a frequency of ≥1% and may serve as biological markers for disease-associated genes 86. Functional polymorphisms that modulate gene expression may underlie interindividual variation in BC susceptibility and clinical outcomes 87. Lo et al. examined SNPs in several mitotic checkpoint genes in 698 primary BC patients and 1,492 healthy controls, and observed that for the SNP rs2910203 in the *PTTG1* gene, carriers of the C1892G genotype had a significantly higher risk of BC 88. Additionally, a combined effect of SNPs in *TTK*,* BUB1B*, and *PTTG1* on BC risk was confirmed through genotype/haplotype analysis 88. Brendle et al. investigated whether SNPs in CIN-related genes affect BC risk and clinical outcomes in a Swedish cohort of BC cases and found that for the SNP rs1862392 in the *PTTG1* gene, carriers of the TA genotype were more likely to have tumors with regional lymph node metastasis compared to carriers of the wild-type genotype 89. These findings suggest that functional polymorphisms in *PTTG1* influence BC susceptibility and progression. Future studies should confirm the efficiency of these SNPs as biomarkers and investigate how they mechanistically regulate securin function.

### Subcellular localization

Accumulating evidence has demonstrated that the subcellular localization of securin also has a prognostic impact on BC. However, inconsistencies remain among the conclusions of relevant studies. Gurvits et al. reported that cytoplasmic securin expression in BC cells was associated with aggressive subtypes and high mortality rates 90. Securin exhibited low (or absent) nuclear expression in benign breast epithelia and luminal carcinomas, whereas HER2-amplified and triple-negative carcinomas showed marked cytoplasmic overexpression 90. Similarly, Repo et al. found that high expression and cytoplasmic localization of securin were directly associated with aggressive tumor features and poorer patient survival in BC 91. In patients with TNBC, cytoplasmic securin localization correlated with a significantly elevated mortality risk and a reduced estimated 5-year survival rate compared to patients with predominantly nuclear securin expression 91. In contrast, another study conducted by Repo et al. in 2020 reported that securin was predominantly localized in the nucleus, though both nuclear and/or cytoplasmic immunoreactivity were occasionally observed in invasive BC 84. Future studies analyzing additional BC samples across different subtypes are needed to clarify the correlation between securin subcellular localization patterns and clinical outcomes. In addition, it has been reported that SPTBN1 mediates the cytoplasmic constraint of securin and impaired its oncogenic activity in human seminoma 92. Considering that SPTBN1 also regulates cell growth and EMT in BC 93, it is imperative to conduct a combined analysis of the expression and subcellular localization of both securin and SPTBN1 in BC.

### DNA methylation

DNA methylation, one of the most widely studied epigenetic modifications, refers to the transfer of a methyl group from S-adenosylmethionine to cytosine residues within CpG dinucleotides by DNA methyltransferases. DNA hypermethylation and hypomethylation are both implicated in the development and prognosis of BC 94. Qi et al. identified aberrantly expressed hub genes potentially regulated by DNA methylation in BC and found that *PTTG1* was one of the 12 hub genes whose promoter was hypomethylated in BC and was accountable for significantly poor clinical outcomes 95. These findings suggest that DNA methylation is potentially a major cause of abnormal expression of *PTTG1* in BC.

## Effects of securin on BC progression

### Cell proliferation, stemness, migration, and invasion

Over the past decades, several studies have shown that securin is associated with BC cell proliferation. Xie and Wang demonstrated that securin overexpression promoted MCF-7 cell proliferation by inducing nuclear exclusion of the cyclin-dependent kinase inhibitor 1B (p27), thereby alleviating p27-mediated G1-phase arrest 96. Khazaei et al. reported that securin knockdown by siRNA inhibited MDA-MB-231 cell proliferation 42. Meng et al. found that securin enhanced BC cell viability and proliferation by regulating cyclin (*CCNA2* and *CCNB2*) expression 51.

Securin also plays a role in regulating the stemness, migration, and invasion of BC cells. Liao et al. reported that knockdown of securin reduced motility, invasion, and metastasis of BC cells by suppressing Rho guanine nucleotide exchange factor-H1 (GEF-H1) expression and RhoA activation. In contrast, overexpression of securin had the opposite effects 97. Yoon et al. reported that securin promoted BC cell migration and invasion through epithelial-to-mesenchymal transition (EMT). This study further revealed that downregulation of securin reduced the self-renewal capacity and tumorigenic potential of BT549 cells, while securin overexpression had the opposite effects. Mechanistically, these effects were partially attributed to AKT activation, a key signaling pathway governing EMT and stemness in cancer cells 40. Yin et al. reported that knockdown of securin markedly inhibited MDA-MB-231 cell invasion and the activities of MMP-2 and MMP-9, while ectopic expression of securin promoted BC cell invasion 41. In the investigation of bisphenol A (BPA), a mass-produced industrial compound implicated in BC progression, Deng et al. observed that BPA exposure significantly promoted BC cell proliferation and migration but not invasion. Subsequent bioinformatic analysis identified securin as one of the key downstream targets of BPA. BPA elevated securin protein level by suppressing miR-381-3p, and securin knockdown attenuated BPA-induced MCF-7 cell proliferation and migration 55. Xing et al. demonstrated that securin acts as a downstream effector of SOS1, modulating cancer stemness and M2 macrophage polarization 49. Li et al. performed single-cell RNA sequencing on 16 paired samples of primary BC and metastatic lymph nodes and observed that dual-positive RAC2/securin BC stem cells, which exhibit the highest stem-like traits, were markedly enriched in metastatic lymph nodes. These cells may drive tumor metastasis by activating metastasis-promoting transcription factors and signaling cascades 98. Future research could focus on the influence of securin on BC cell cycle regulation and the effects of its post-translational modifications on cell proliferation, stemness, migration, and invasion.

### Apoptosis

Contradictory evidence persists regarding securin's regulation of apoptotic pathways in BC cells. Yu et al. demonstrated that securin overexpression induced p53-dependent apoptosis in MCF-7 cells 71. Similarly, Hamid and Kakar reported that securin overexpression elevated *Bax* expression and triggered apoptosis through *p53* in MCF-7 cells 99. However, Xie and Wang found that knockdown of securin did not alter apoptosis in MCF-7 cells 96.

Several possibilities could account for these contradictions. For instance, functional redundancy within the *PTTG* gene family may enable compensatory mechanisms, where paralogous genes maintain apoptotic homeostasis when securin is silenced. In addition, the efficiency of the shRNA used for securin knockdown must be systematically evaluated, as insufficient knockdown may fail to reach the threshold required to impact apoptosis. Alternatively, supraphysiological overexpression of securin might induce non-specific cellular stress responses, leading to cell death. Collectively, further investigations across diverse BC cell lines under varying experimental conditions are warranted to clarify the influence of securin on cell apoptosis.

### Senescence

**Figure 3:**
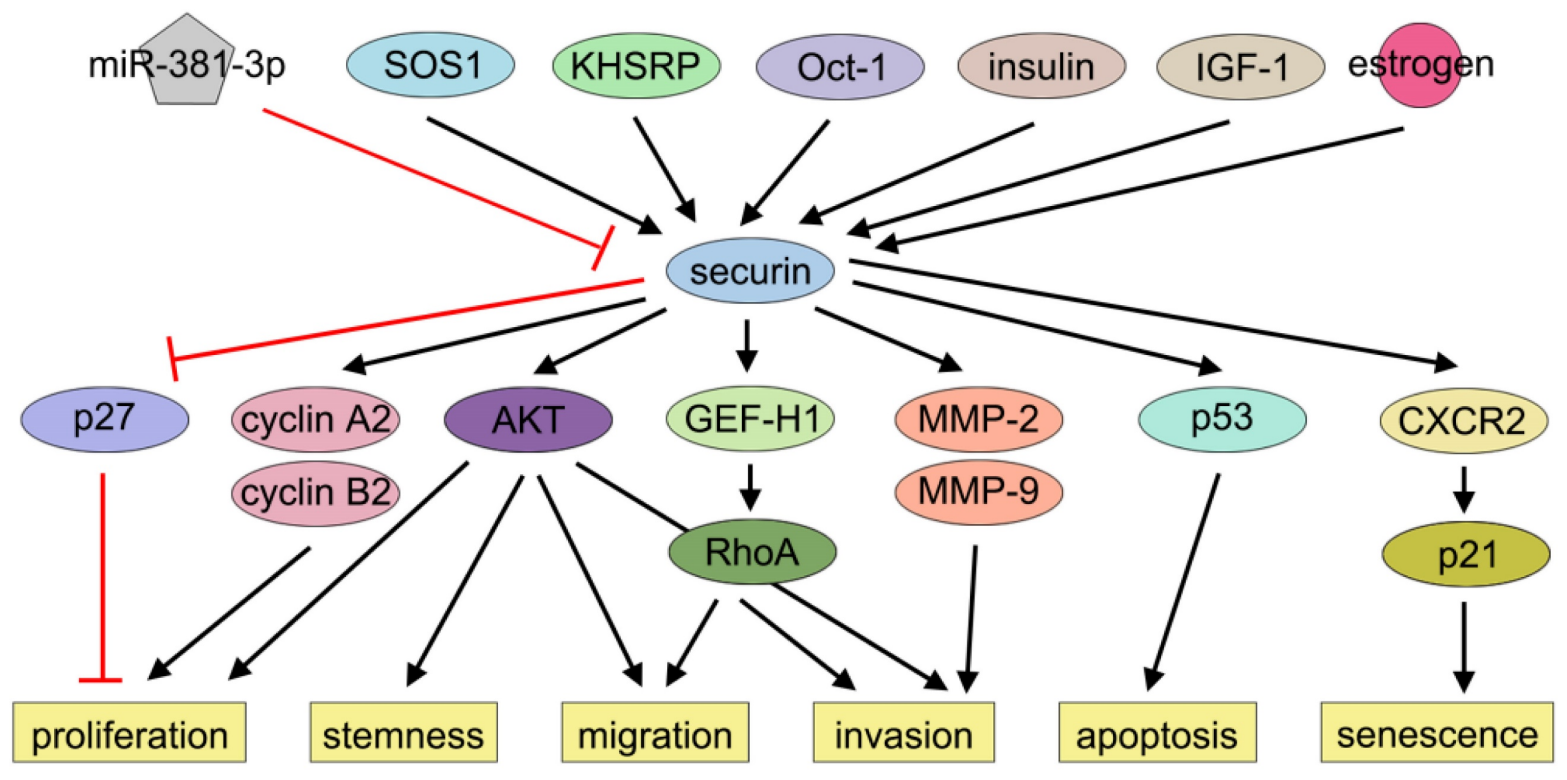
** Effects of securin on BC progression.** Securin exerts multiple effects, such as proliferation, migration, invasion, and apoptosis via many signaling pathways in BC.

Cellular senescence, a state of prolonged cell cycle arrest, is considered an important tumor-suppressive mechanism 100, 101. Chen et al. and Yu et al. demonstrated that securin played a crucial role in determining radiosensitivity and cell fate. Radiation induced apoptosis in wild-type cells but induced senescence in securin-null cells. Moreover, the knockdown of securin switched irradiation-induced apoptosis to senescence in MDA-MB-231 cells 102, 103. Ruan et al. reported that securin overexpression reinforced senescence through CXCR2 signaling, while escape from CXCR2/p21-dependent senescence surveillance was essential for securin's oncogenic effects 104 (Figure 3). Thus, securin acts as a key regulator of radiation-induced cell fate decisions in BC. More studies need to elucidate how securin controls the apoptosis-senescence transition following irradiation and how this pathway can be therapeutically modulated.

### Chemosensitivity and resistance

Studies have confirmed that securin is associated with sensitivity or resistance to multiple anticancer drugs. Ghayad et al. explored new biomarkers of endocrine resistance in ERα-positive BC and found that *PTTG1* was significantly overexpressed in samples from patients who relapsed after tamoxifen treatment compared to those who did not. Furthermore, *PTTG1* was identified as an independent prognostic marker correlated with significantly shorter RFS 105. Yu et al. reported that gefitinib, a tyrosine kinase inhibitor targeting EGFR, downregulates securin at the protein level by reducing its stability without affecting gene transcription. Securin overexpression increased both proliferation rates and resistance to gefitinib-induced death in several cancer cell lines, indicating that high securin levels may confer cellular resistance to gefitinib 106. Yin et al. demonstrated that ectopic expression of securin partially reversed simvastatin-mediated inhibition of cell invasion in MDA-MB-231 cells 41. Bashari et al. investigated candidate genes associated with drug resistance and susceptibility in TNBC using data from PharmacoDB and found that high expression levels of *PTTG1* were significantly correlated with sensitivity to IKK 16 and bromopyruvic acid 44. Targeted strategies for inhibiting securin transcription or decreasing protein stability in conjunction with EGFR inhibitors or tamoxifen may address both the underlying mechanisms and translation opportunities against securin-mediated chemoresistance.

### Adverse reactions following radiotherapy

The relationship between *PTTG1* and adverse reactions after radiotherapy has produced conflicting conclusions. Suga et al. identified haplotypes of SNPs associated with the risk of early adverse skin reactions (EASRs) after radiotherapy in 399 Japanese patients with BC, and found that the GTTG haplotype in *PTTG1* was associated with a significantly reduced risk of EASRs 107. Aguiar et al. evaluated the association between SNPs and acute radiation dermatitis (RD) in patients with BC and found that four SNPs in *PTTG1* were associated with RD 108. However, Murray et al. and Mumbreker et al., analyzed distinct cohorts of BC patients, reported no significant association between SNPs in the *PTTG1* gene and EASRs following radiotherapy 109, 110. Discrepancies among these findings could be attributed to limited sample sizes and geographic heterogeneity among patient populations. Nevertheless, a general consensus has emerged that complex clinical manifestations, such as EASRs, arise from the interplay of multiple molecular pathways and pathophysiological mechanisms. Therefore, further studies are needed to clarify the connection between genetic variations in *PTTG1* and adverse reactions following radiotherapy in BC patients.

## Conclusions

In this review, we systematically examined the multiple roles of securin in BC. While substantial progress has been made over the past decades in delineating securin's involvement in the regulation of CIN and BC progression, several uncharted areas demand further exploration to fully realize its diagnostic, prognostic, and therapeutic potential. Future investigations should rigorously characterize disparities in the pathobiological role of securin across geographically diverse populations and molecular subtypes of BC, with particular attention to variations in genetic backgrounds, environmental exposures, and healthcare disparities that may influence its oncogenic functions, therapeutic responses, and prognostic significance. Additionally, expanded studies focusing on the pharmacogenomic landscape of securin, especially its role in modulating therapeutic sensitivity and resistance, will be valuable. SECURA-3, a novel RNA aptamer targeting securin developed in 2020, displayed high detection sensitivity in MCF-7 and HeLa cells 111. Future studies can focus on exploiting its diagnostic potential and evaluating its therapeutic applications.

Elucidating the multidimensional protein interactome through which securin governs CIN—including separase regulation, DNA damage response coordination, and cell cycle checkpoint control—is equally critical for clarifying its roles in BC progression. To this end, integrating high-throughput interactome screening, multi-omics profiling, and single-cell sequencing approaches may provide key insights. As targeting CIN gains increasing therapeutic momentum, decoding securin's interaction network holds promise for breakthroughs in BC management. Furthermore, the generation of tissue-specific *Pttg1* knockout or transgenic mouse models is necessary to better elucidate the physiological and pathological roles of securin in the mammary gland.
